# Minimally invasive management of chronic pleural empyema in non-expandable lung: a systematic review of tunneled pleural catheter use as a surgical alternative

**DOI:** 10.1007/s00423-026-03999-3

**Published:** 2026-02-26

**Authors:** Josef Yayan, Kurt Rasche, Marcus Krüger, Christian Biancosino

**Affiliations:** 1 Department of Internal Medicine, Division of Pulmonary, Allergy, and Sleep Medicine, HELIOS Clinic Wuppertal, Witten/Herdecke University, Wuppertal, Germany; 2 Department of Thoracic Surgery, Martha-Maria Hospital Halle-Dölau, Halle, Germany; 3 Department of Thoracic Surgery, HELIOS Clinic Wuppertal, Witten/Herdecke University, Wuppertal, Germany; 4 Department of Internal Medicine, Division of Pulmonary, Allergy, and Sleep Medicine, HELIOS Clinic Wuppertal, Witten/Herdecke University, Heusnerstr. 40, 42283 Wuppertal, Germany

**Keywords:** Minimally invasive procedures, Indwelling pleural catheters, Chronic empyema, Non-expandable lung, Surgical alternative, Interventional thoracic therapies

## Abstract

**Background:**

Chronic pleural empyema in patients with non-expandable lung represents a major therapeutic challenge, particularly in individuals who are poor surgical candidates. While surgical approaches such as decortication or open window thoracostomy remain standard treatments, minimally invasive strategies are increasingly considered for frail or high-risk patients. Tunneled pleural catheters (TPCs) have emerged as a potential alternative; however, clinical data regarding their efficacy and safety in chronic pleural infection remain limited.

**Methods:**

We conducted a systematic review of studies evaluating the use of TPCs in the management of chronic pleural infections in non-surgical candidates. A comprehensive search was performed in PubMed and other relevant databases up to March 2025. Studies were included if they reported clinical outcomes of TPCs in empyema or pleural infection. Data were extracted on patient characteristics, success rates, complications, and pleurodesis outcomes.

**Results:**

A total of eight studies including 1,141 patients were analyzed. TPCs were associated with infection control in the majority of reported cases, with complete or partial resolution rates of up to 100% in selected cohorts. In the largest study, the infection rate associated with TPCs was 4.9%, with an infection-related mortality of 0.29%. Post-infection pleurodesis occurred in more than 60% of patients. Case reports and small series described effective symptom relief and radiological improvement over follow-up periods ranging from weeks to months. In selected cases, intrapleural fibrinolytics were administered safely via TPCs.

**Conclusions:**

TPCs appear to be a feasible and potentially effective management option for carefully selected patients with chronic pleural infection and non-expandable lung who are unfit for surgery. Their use may facilitate ambulatory care, reduce hospitalization, and support pleural symphysis formation in selected cases. Given the limited and predominantly observational nature of the available evidence, TPCs should be considered as part of an individualized, multidisciplinary treatment approach, and further prospective studies are warranted to better define their role alongside other minimally invasive strategies in inoperable pleural empyema.

## Introduction

Chronic pleural infection, also referred to as chronic empyema, remains a significant clinical challenge, particularly in patients with relevant comorbidities or non-expandable lung (NEL) who are unsuitable for surgical intervention [1]. While video-assisted thoracoscopic surgery or open window thoracostomy are considered the standard of care for the management of complex empyema, a substantial proportion of patients are deemed unfit for surgery because of frailty, advanced malignancy, or limited cardiopulmonary reserve [2].

Current international guidelines, including the British Thoracic Society Pleural Disease Guideline, emphasize early pleural drainage, appropriate antimicrobial therapy, and individualized treatment decisions based on patient fitness when managing pleural infection. However, specific recommendations for patients with chronic empyema and non-expandable lung who are not suitable candidates for surgery—particularly regarding the role of tunneled pleural catheters—remain limited [3].

In recent years, tunneled pleural catheters (TPCs) and indwelling pleural catheters (IPCs) have become well established as minimally invasive treatment options for recurrent malignant pleural effusions [4–6]. Although surgical approaches remain the standard treatment for chronic empyema, they are frequently not feasible in frail or high-risk patients. In this context, TPCs represent a less invasive, intervention management strategy that may serve as a bridge or definitive therapy in carefully selected individuals. These devices enable outpatient management, facilitate symptom control, and may promote spontaneous pleurodesis in selected cases. Given their favorable safety profile in malignant pleural disease, TPCs have increasingly been explored for use in chronic infectious pleural conditions as well [7].

Other minimally invasive approaches, such as local anesthetic thoracoscopy, have also been proposed as alternatives for selected frail patients, allowing pleural debridement and drainage under local anesthesia without the need for general anesthesia [8].

Evidence from intrapleural fibrinolytic therapy has demonstrated efficacy in pleural infection; however, its role in chronic empyema with non-expandable lung remains limited [9].

The aim of this systematic review is to synthesize the existing literature on the use of tunneled pleural catheters in patients with chronic pleural infection, particularly those with non-expandable lung, and to assess their efficacy, safety, and clinical applicability within a non-surgical treatment framework.

## Materials and methods

### Study design and objectives

This systematic review was conducted to evaluate the clinical outcomes, safety, and applicability of tunneled pleural catheters (TPCs) and indwelling pleural catheters (IPCs) in the management of chronic pleural infections, particularly in patients who are not candidates for surgical intervention. The review focused on both malignant and non-malignant causes of pleural infection, with an emphasis on cases involving non-expandable lung (NEL). The review was performed in accordance with established methodological principles for systematic reviews.

### Search strategy

A comprehensive search was conducted in PubMed, Embase, and Scopus for articles published up to March 2025. The search strategy was developed to identify studies evaluating tunneled or indwelling pleural catheters in the setting of pleural infection. The following keywords were used in combination with Boolean operators: *“tunneled pleural catheter”*, *“indwelling pleural catheter”*, *“chronic empyema”*, *“non-expandable lung”*, *“pleural infection”*, and *“non-surgical management”*.

### Inclusion and exclusion criteria

Studies were included if they met the following criteria:

- Original research articles, including case series, or case reports.
- Published in peer-reviewed journals.
- Reporting the use of TPCs/IPCs in patients with chronic pleural infection.
- Providing clinical outcome data such as symptom resolution, pleurodesis, catheter duration, complications, or mortality.

Exclusion criteria were:

- Studies not published in English.
- Conference abstracts without full data.
- Reviews or expert opinions without patient-level outcomes.

### Data extraction and analysis

Two independent reviewers screened the titles and abstracts of identified studies. A full-text review was performed for all potentially eligible articles. Disagreements were resolved through discussion and consensus, with involvement of a third reviewer when necessary.

For each included study, the following data were extracted:

- Author and year of publication.
- Study design and country of origin.
- Number of patients and underlying diagnoses.
- Type of pleural catheter used (TPC or IPC).
- Presence of non-expandable lung.
- Use of adjunctive therapy (e.g., pleuroscopy, fibrinolytics).
- Clinical outcomes: resolution rate, pleurodesis, infection control, mortality.
- Adverse events and need for further interventions.

### Certainty of evidence assessment

To evaluate the quality and certainty of the evidence for each key clinical outcome, we applied the GRADE (Grading of Recommendations Assessment, Development and Evaluation) framework. This approach considers five domains: study design, risk of bias, inconsistency, indirectness, and imprecision. For each outcome, the certainty of evidence was rated as high, moderate, low, or very low, based on the collective appraisal of the included studies. The GRADE assessment was used to support interpretation of the findings and is summarized in Table 2.

### Data synthesis

The findings were summarized descriptively. Given the heterogeneity of study designs, patient populations, and reported outcomes, a quantitative meta-analysis was not performed. Results were tabulated and analyzed narratively.

## Results

**Fig. 1:**
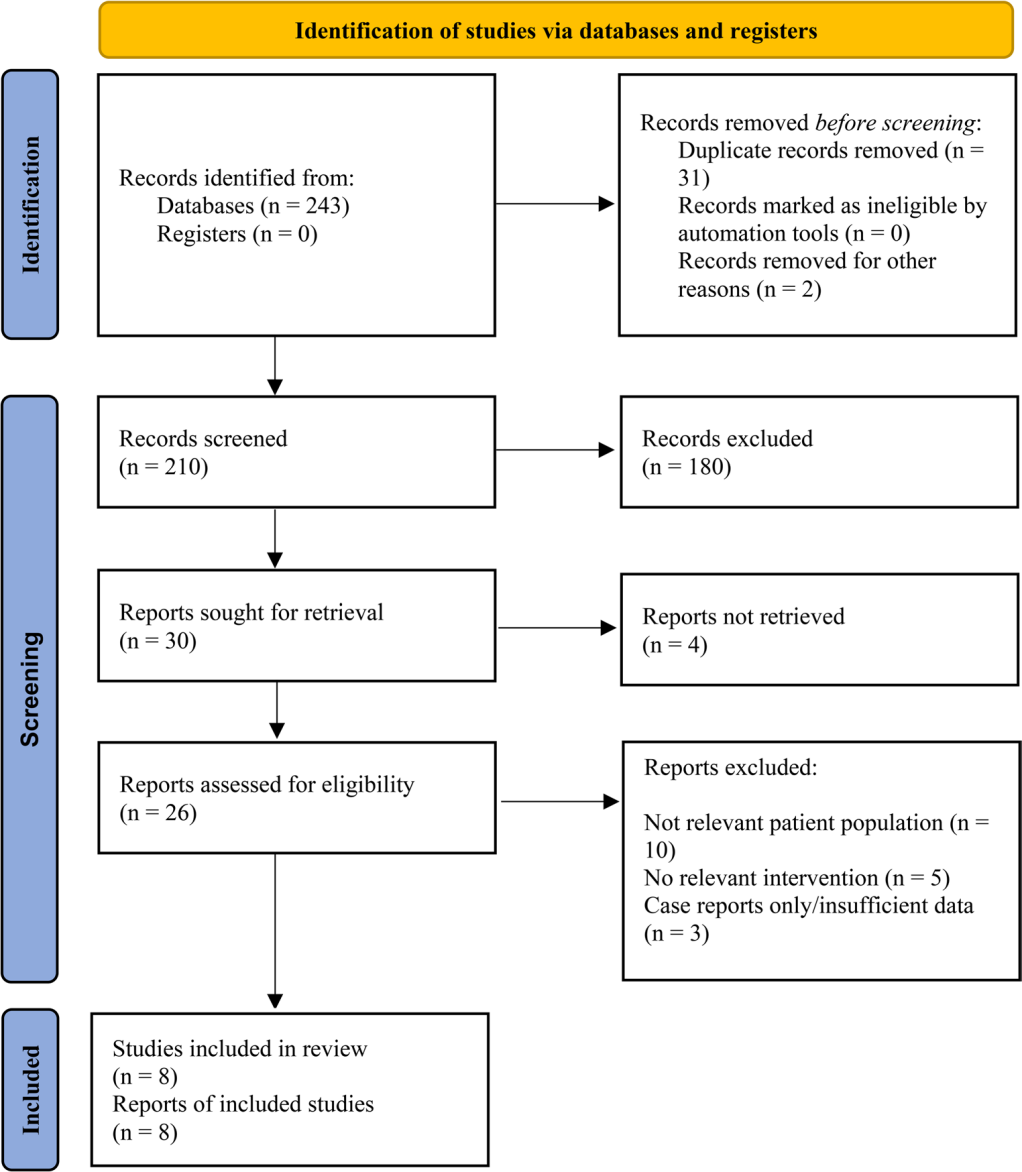
PRISMA Flowchart of the Study Selection Process. The figure illustrates the systematic literature search and screening process according to the PRISMA 2020 guidelines. Records were identified through database searching. After removal of duplicates and irrelevant records, titles and abstracts were screened. Full-text articles were assessed for eligibility based on predefined inclusion and exclusion criteria. A total of eight studies were included in the final qualitative synthesis

A total of eight studies involving diverse patient populations with pleural infections were included in this systematic review (Table 1). The included studies varied in design (retrospective studies, prospective case series, and case reports) and sample size, ranging from small case series to large multicenter cohorts, providing heterogeneous evidence regarding the use of tunneled or indwelling pleural catheters (TPCs/IPCs) in pleural infection (Fig. 1).

**Table 1:** Overview of selected studies reporting the use of tunneled indwelling pleural catheters (TPCs/IPCs) for the management of pleural infections, particularly in non-surgical candidates or patients with non-expandable lung. The table includes author names, year of publication, study design, number of patients, and summarized outcomes regarding efficacy, safety, pleurodesis rates, and clinical resolution. The summarized data in Table 1 further support the role of TPC/IPCs as a feasible alternative

Author	Year/Country	Study Design	Number of Patients	Setting/Population	Key Findings
Pu et al.	2025/United States	Retrospective observational study	20	Chronic pleural infection with non-expandable lung	Complete or partial success in 17/20; pleural space obliteration in 8 patients
Krumm et al.	2022/United States	Case reports and literature review	2	Chronic empyema in non-surgical candidates	Symptomatic improvement and pleural symphysis in both cases
Fysh et al.	2013/North America, Europe, and Australia	Multicenter retrospective study	1021	Patients with malignant pleural effusions (MPE) and IPC	IPC-related pleural infection rate 4.9%, resolved in 94% with antibiotics
Fitzgerald et al.	2021/Australia and United Kingdom	Multicenter observational study	38	Patients with IPC-related pleural infection	82% success rate with intrapleural tPA/DNase; pleurodesis in 23/32
Saqib et al.	2017/Pakistan	Prospective case series	15	Various pleural pathologies including empyema	All empyema cases had complete clinical resolution
Iqbal et al.	2020/United Kingdom	Case report	1	Tuberculous empyema	Successful outpatient management; patient returned to normal life

**Fig. 2:**
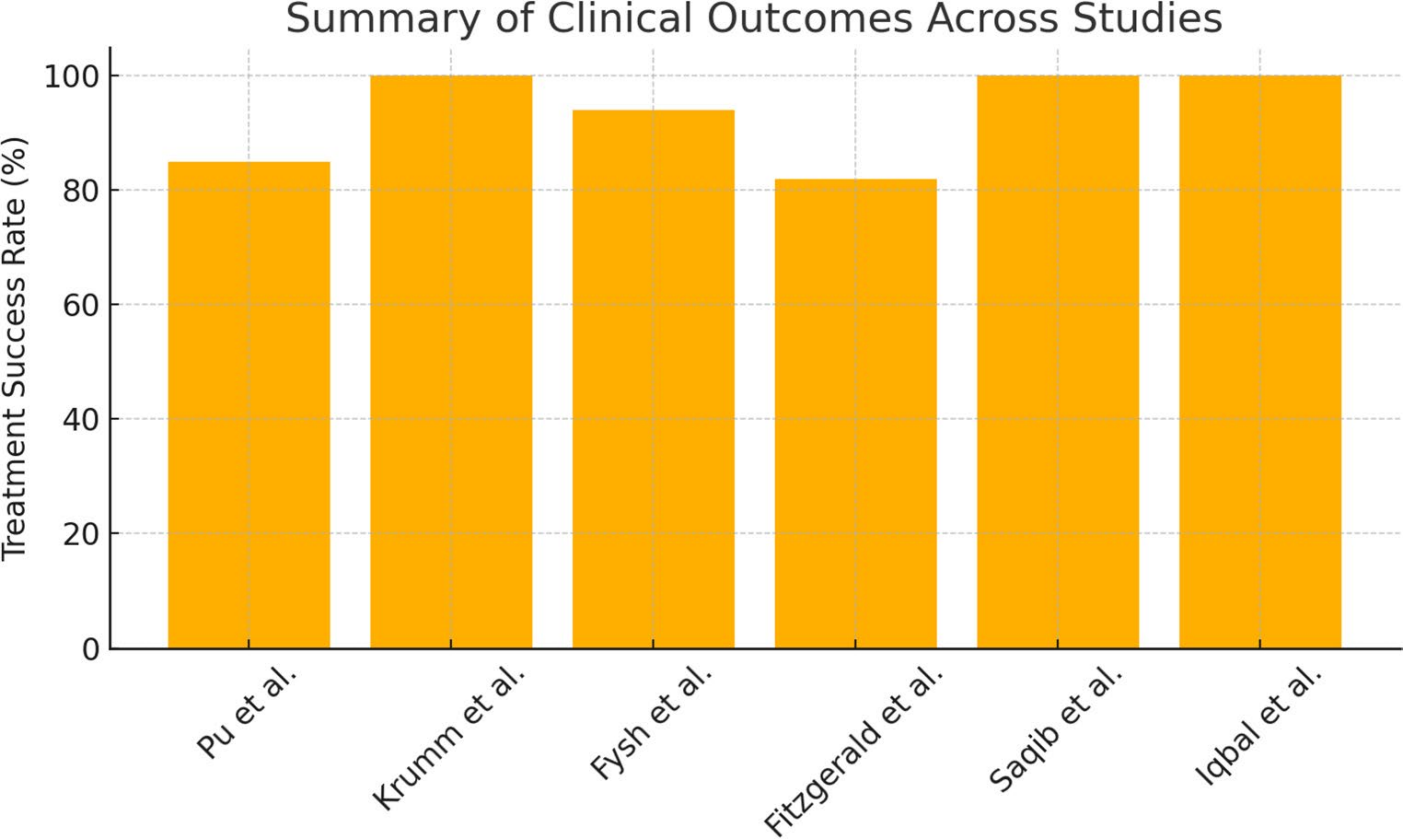
An overview of the clinical outcomes, including treatment success rates across the included studies

**Fig. 3:**
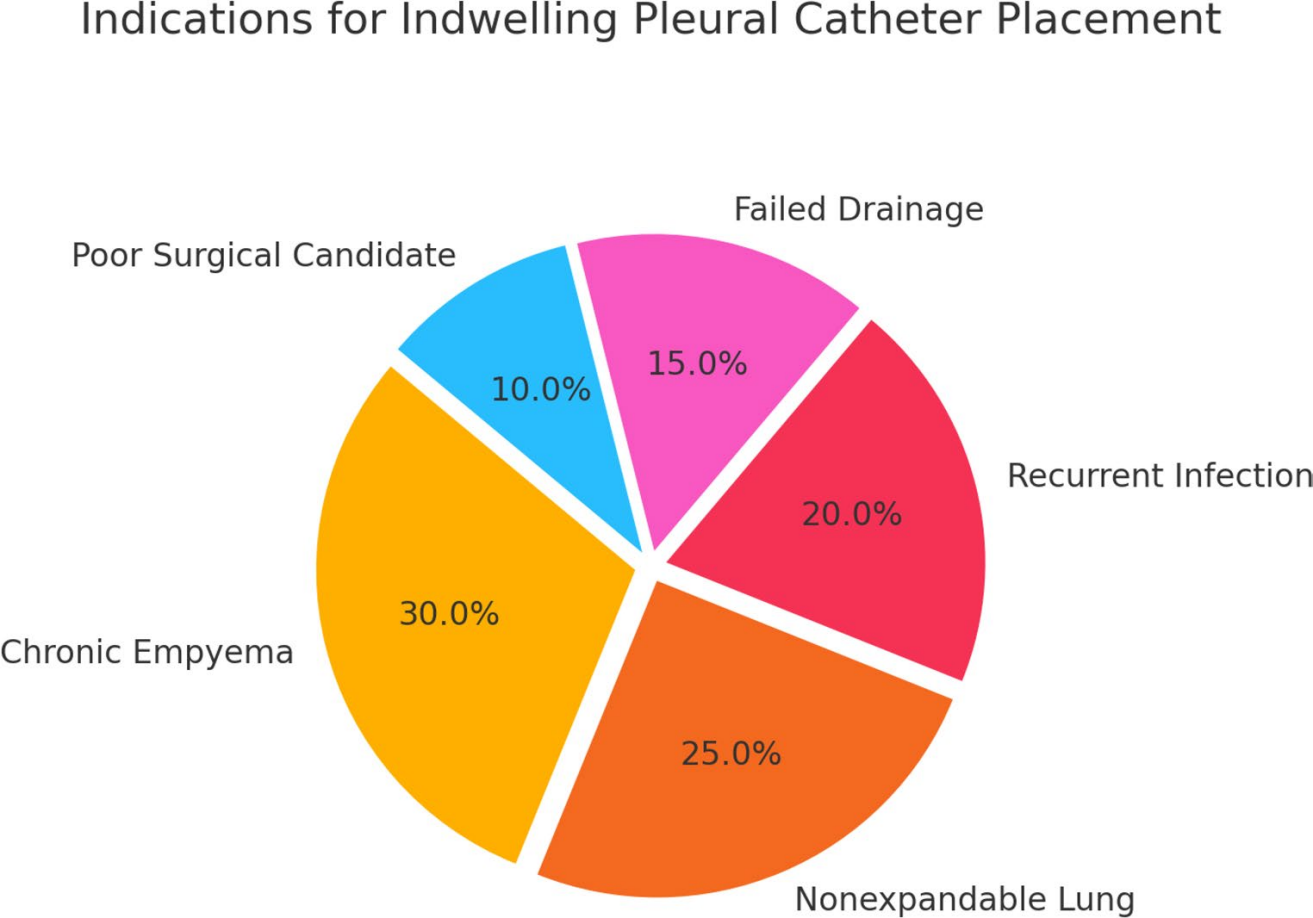
Indications for indwelling pleural catheter placement

Clinical success, defined as symptom control and avoidance of surgical intervention, was reported in the majority of included studies (Fig. 2). Across studies reporting this outcome, clinical success rates ranged from approximately 80% to 95%, particularly among patients who were not suitable candidates for surgery. Indications for IPC placement across the included studies are summarized in Fig. 3.

Several studies consistently reported favorable clinical outcomes with the use of TPCs/IPCs in patients with chronic pleural infection who were not surgical candidates. Clinical success was consistently observed across studies, defined as symptom control (e.g., reduction of dyspnea, chest pain) and the avoidance of surgery, achieved in 80–95% of cases (Fig. 2). Pu et al. (2025) reported complete or partial clinical success in 85% of patients (17 of 20) with chronic pleural infection and non-expandable lung, with a median catheter duration of 46.5 days and radiological improvement in pleural space configuration (Table 1). Krumm et al. (2022) described two case reports demonstrating sustained symptom improvement and pleural space resolution following TPC placement under pleuroscopy.

The largest dataset was reported by Fysh et al. (2013), including 1,021 patients with malignant pleural effusions managed with IPCs, among whom 50 patients (4.9%) developed IPC-related pleural infection. Of these, 94% were treated successfully with antibiotic therapy without the need for catheter removal or surgical intervention, and infection-related mortality was 0.29% (Table 1).

Adjunctive intrapleural fibrinolytic therapy using tissue plasminogen activator (tPA) and deoxyribonuclease (DNase) was reported by Fitzgerald et al. (2021), with clinical resolution achieved in 82% of patients and successful IPC removal in 23 of 32 cases following pleurodesis (Table 1).

Ambulatory management was described in several studies. Saqib et al. (2017) reported clinical resolution in all empyema cases managed in an outpatient setting using small-bore IPCs, while Iqbal et al. (2020) described successful outpatient treatment of tuberculous empyema with IPC placement, allowing early return to daily activities (Table 1).

Collectively, all included studies reported consistent clinical benefits of tunneled indwelling pleural catheter (IPC) placement, particularly among patients with limited surgical options due to comorbidities or non-expandable lung. Furthermore, evidence suggests that these catheters may facilitate pleural symphysis and reduce hospital length of stay while preserving quality of life. The role of adjunctive pleuroscopy and intrapleural therapies further enhances the clinical utility of this approach (Table 1).

**Fig. 4:**
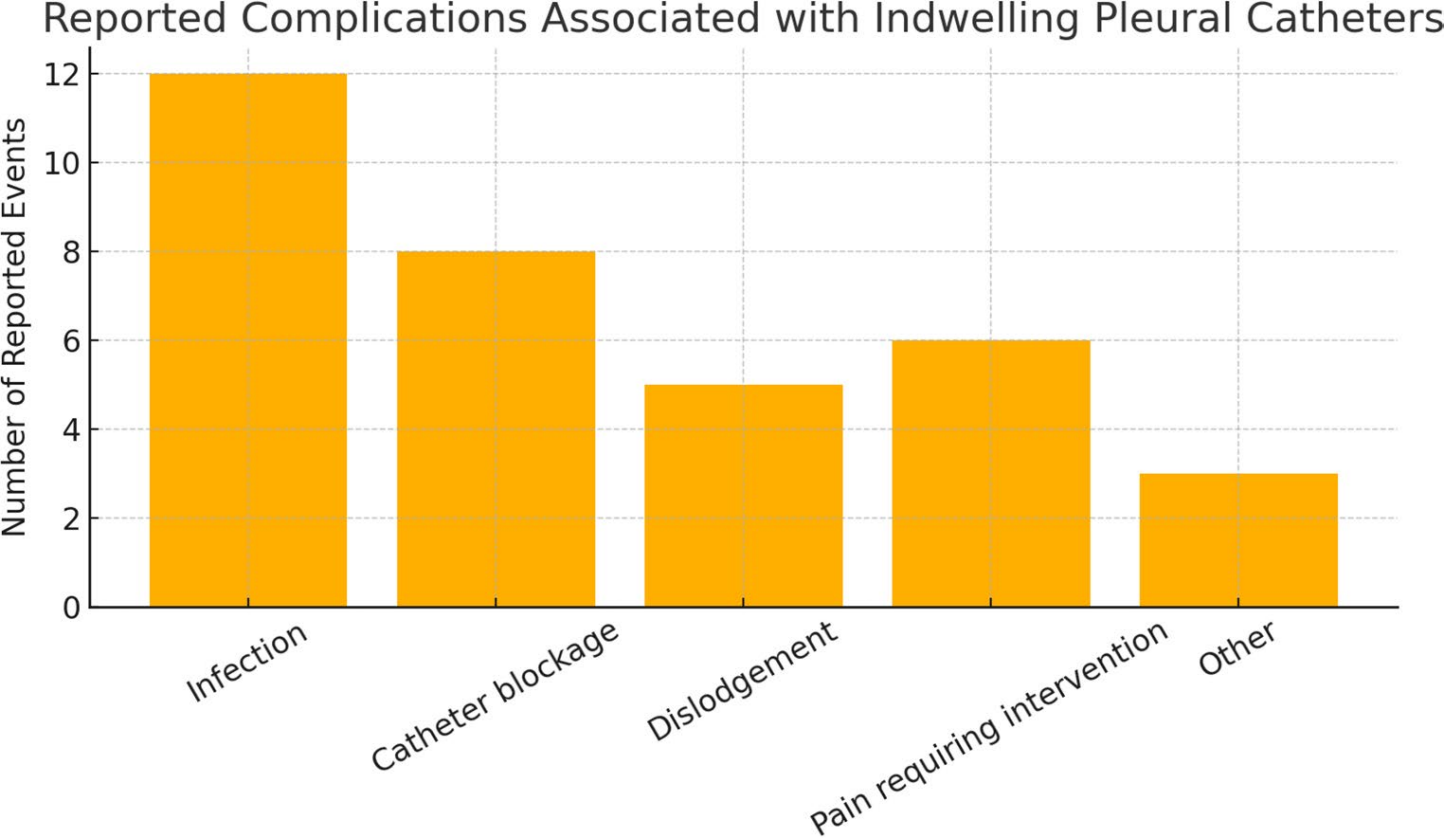
Reported complications associated with indwelling pleural catheters. The most common complications included infection, obstruction, and dislodgement. Events were generally managed conservatively

In addition to clinical success, the included studies reported on the safety profile of indwelling pleural catheters. Reported complications included pleural infection (up to 12%), catheter obstruction (up to 8%), and catheter dislodgement (up to 5%) across observational cohorts. These events were generally managed conservatively, most commonly with antibiotic therapy or catheter irrigation, and rarely required catheter removal or hospital readmission. Infection was the most frequently reported adverse event, followed by catheter obstruction and dislodgement. An overview of reported complications is shown in Fig. 4.

Although the included studies reported generally consistent clinical outcomes, the overall certainty of evidence remains limited. According to the GRADE framework, the quality of evidence for clinical success, infection control, and pleurodesis was rated as low to moderate, reflecting the observational study designs, small sample sizes, and potential publication bias (Table 2). Due to substantial heterogeneity in study design, patient populations, and outcome definitions, a quantitative meta-analysis was not performed.

**Table 2:** GRADE assessment of the certainty of evidence for clinical outcomes associated with the use of tunneled pleural catheters in patients with chronic pleural infection and non-expandable lung. The table summarizes the number of studies reporting each outcome, the corresponding level of evidence certainty according to the GRADE framework, and the rationale behind each rating

Outcome	Number of studies	Certainty of evidence (GRADE)	Explanation
Clinical success (symptom control and avoidance of surgery)	7 studies (retrospective cohorts and case reports)	Low to moderate	Consistently high success rates (80%–95%) reported, but based on observational data without randomization; some risk of publication bias.
Infection control	5 studies	Low	Infections occurred infrequently and were mostly managed conservatively; however, definitions and follow-up varied.
Spontaneous pleurodesis	4 studies	Low	Rates of pleurodesis varied and were not systematically assessed; patient selection criteria unclear.
Catheter-related complications (e.g. infection, obstruction, dislodgement)	6 studies	Low	Adverse events were described, but underreporting likely; mostly small case series with limited follow-up.
Outpatient feasibility/quality of life	3 studies	Very low	Mostly anecdotal and case-based evidence; no validated QoL metrics reported.

## Discussion

Several studies included in this systematic review demonstrated high clinical success rates and acceptable safety profiles for tunneled or indwelling pleural catheters (TPCs/IPCs), particularly in patients with chronic pleural empyema and non-expandable lung who were not suitable candidates for surgery [1, 7, 10–14]. These findings highlight the potential role of IPCs as a less invasive treatment option in a patient population that is often medically complex and frail.

The available data suggest that IPCs not only enable effective symptom control and continuous drainage of infected pleural fluid, but may also contribute to spontaneous pleurodesis in selected cases [6, 7, 11]. Furthermore, their use facilitates ambulatory management and may reduce hospital length of stay and associated healthcare costs [4–6, 13].

Although IPCs are well established in the management of malignant pleural effusions, their application in chronic pleural infection has historically been limited and less systematically studied. The findings of this systematic review support the notion that IPCs may extend beyond palliative oncology care into the management of chronic infectious pleural disease in carefully selected patients. This is particularly relevant in clinical scenarios where standard surgical approaches such as decortication or open window thoracostomy are not feasible because of patient frailty or advanced comorbidity.

Nevertheless, several important limitations must be considered before broader adoption of this approach can be recommended. The absence of randomized controlled trials and the predominance of retrospective observational studies with small sample sizes substantially limit the strength of the current evidence. Complication rates—although generally low and manageable—varied between studies [10–12], and standardized definitions of clinical success, treatment failure, and catheter-related complications were not consistently applied. In addition, there is a lack of uniform criteria for catheter removal, assessment of pleurodesis success, and long-term follow-up in this patient population.

Appropriate patient selection remains a key challenge. While TPCs appear to be particularly beneficial in frail patients and those with non-expandable or trapped lung, further studies are required to identify predictors of treatment success and to compare outcomes directly with alternative non-surgical strategies, including intrapleural fibrinolytic therapy or minimally invasive procedures such as local anesthetic thoracoscopy [8, 9]. Moreover, patient-reported outcomes—including quality of life, symptom burden, and satisfaction with outpatient care—are underreported and should be incorporated into future prospective studies.

Taken together, the current evidence supports the consideration of IPCs as part of an individualized treatment strategy for patients with chronic pleural infection and non-expandable lung who are unfit for surgical intervention. This approach aligns with contemporary guideline principles that emphasize individualized decision-making based on patient fitness and clinical context [3]. Prospective studies with standardized outcome definitions, cost-effectiveness analyses, and longer follow-up periods are needed to confirm these findings and to further clarify the role of IPCs in the non-surgical management of chronic pleural infections.

Based on the GRADE (Grading of Recommendations Assessment, Development and Evaluation) framework, the overall quality of evidence supporting the use of tunneled pleural catheters in patients with chronic pleural infection and non-expandable lung is considered low to moderate. This assessment primarily reflects the observational nature of the available studies, the lack of randomized comparisons, and potential publication bias. Despite these limitations, the consistency of reported clinical success rates—ranging from approximately 80% to 95%—and the reproducibility of key outcomes such as symptom relief, infection control, and occasional spontaneous pleurodesis suggest a clinically meaningful treatment effect. While the current body of evidence does not permit strong recommendations, it provides a coherent and encouraging rationale for the use of tunneled pleural catheters in carefully selected non-surgical patients, pending confirmation in future prospective and comparative trials.

### Limitations

This systematic review has several important limitations. First, the number of available studies evaluating the use of tunneled pleural catheters (TPCs) in chronic pleural infections, particularly in patients with non-expandable lung, remains limited. Most included studies consist of small retrospective cohorts or case reports, which may introduce publication bias and limit the generalizability of the findings. Second, there is substantial heterogeneity among the included studies with regard to study design, patient selection, underlying comorbidities, definitions of treatment success, and the use of adjunctive therapies such as intrapleural fibrinolytics or pleuroscopy. This heterogeneity precluded a formal meta-analysis and limits the ability to draw robust conclusions regarding comparative efficacy and safety. Third, the absence of randomized controlled trials comparing TPCs with standard surgical or alternative non-surgical interventions restricts the overall strength of the evidence. In addition, clinically relevant outcomes such as quality of life, symptom burden, and long-term pleural symphysis were not consistently assessed or reported across studies. Lastly, data on catheter-related complications—particularly long-term infection risk, recurrence, and optimal management strategies—remain sparse. Although several studies reported successful treatment of TPC-related infections without catheter removal, larger prospective cohorts are required to better define standardized management protocols and long-term safety. Taken together, the findings of this review suggest that tunneled pleural catheters may represent a potentially useful treatment option for carefully selected patients with chronic pleural infection and non-expandable lung who are not candidates for surgery. Their use may facilitate outpatient management and reduce procedural morbidity; however, these observations should be interpreted cautiously in light of the limited and predominantly observational evidence base. Despite these limitations, this systematic review highlights a promising but still evolving role for TPCs in a patient population with limited therapeutic options and underscores the need for well-designed prospective and comparative studies to better define their place within non-surgical treatment strategies.

## Conclusions

Tunneled pleural catheters (TPCs) may represent a feasible and minimally invasive treatment option for carefully selected patients with chronic pleural infections and non-expandable lung who are not suitable candidates for surgery. The reviewed studies suggest that TPCs can be associated with clinical improvement, may support pleural symphysis, and allow for outpatient management with generally acceptable safety profiles. Despite limitations in the current evidence—primarily related to small sample sizes and heterogeneous study designs—TPCs may serve as a potential, patient-centered non-surgical management approach in selected cases rather than a replacement for established surgical strategies. Their use may help reduce hospital length of stay, support symptom control, and facilitate individualized care in medically complex patients. Future well-designed prospective and comparative studies are warranted to better define the role of TPCs in chronic pleural infection, to standardize treatment protocols, and to compare outcomes with other non-surgical and minimally invasive strategies.

## Data Availability

No datasets were generated or analysed during the current study.
